# Foxc2 and BMP2 Induce Osteogenic/Odontogenic Differentiation and Mineralization of Human Stem Cells from Apical Papilla

**DOI:** 10.1155/2018/2363917

**Published:** 2018-07-25

**Authors:** Wen Zhang, Xiaolei Zhang, Junyuan Li, Jianmao Zheng, Xiaoli Hu, Meng Xu, Xueli Mao, Junqi Ling

**Affiliations:** ^1^Guangdong Province Key Laboratory of Stomatology, Guanghua School and Hospital of Stomatology, Sun Yat-sen University, Guangzhou, Guangdong 510055, China; ^2^The Medical Centre of Stomatology, The 1st Affiliated Hospital of Jinan University, Guangzhou 510630, China

## Abstract

As a transcription factor regulated by bone morphogenetic protein 2 (BMP2), Forkhead c2 (Foxc2) plays a pivot role in osteogenesis/odontogenesis. However, the role of Foxc2 and BMP2 in regulating osteo-/odontogenic differentiation and mineralization of stem cells from apical papilla (SCAP) is still uncertain. In this research, overexpression of Foxc2 gene significantly improved the proliferation of SCAP four days and eight days after transfection, but overexpression of both Foxc2 and BMP2 genes significantly inhibited the proliferation of SCAP eight days after transfection. RT-qPCR and western blot results indicated that SCAP-Foxc2-BMP2 significantly upregulated osteo-/odontogenic genes and proteins at most of the time points in SCAP after transfection. Moreover, SCAP-Foxc2-BMP2 formed notably more alkaline phosphatase-positive and alizarin red-positive mineralized nodules than other three group cells sixteen days after transfection. In conclusion, our findings revealed that Foxc2 and BMP2 synergistically promoted osteo-/odontogenic differentiation and mineralization of SCAP *in vitro*.

## 1. Introduction

How to effectively regenerate dentin and repair tooth defects is the key problem in dental regenerative medicine [1]. Tissue engineering dentin is the use of a combination of cells, engineering and material methods, and suitable biochemical and physicochemical factors to regenerate biological dentin. Human stem cells from apical papilla (SCAP) are considered to be suitable seed cells for dentin regeneration, because they have osteo-/odontogenic differentiation potential, good individual specificity, and high proliferative activity [2]. Moreover, they can regularly be obtained from extracted immature third molars or orthodontic teeth in clinical practice [3]. However, without induction, the capability of forming mineralized structure/tissue by SCAP is insufficient [4–6]. Thus, it is necessary to explore the key genes in regulating osteo-/odontogenic differentiation of SCAP and on this basis enhance their expression in order to enable reparative dentin formation.

Dentin development is the result of interaction between dental epithelium and mesenchyme, and multiple molecular pathways are involved in regulating this process [7]. Bone morphogenetic protein (BMP) family, a part of the transforming growth factor beta (TGF-*β*) superfamily, is crucial in dental papilla growth and development [7, 8]. BMP2 is one of the strongest osteo-/odontogenic induction members in the BMP family, supporting osteo-/odontogenic differentiation of dental pulp stem cells [4, 6, 9]. Wu et al. reported that BMP2-containing medium could promote SCAP to form dentin-like tissue [10]. In our previous studies [4, 6], BMP2 gene-modified SCAP (SCAP-BMP2) presented significantly upregulated osteo-/odontogenic differentiation genes/proteins. Thus, for osteogenic or odontogenic differentiation of SCAP, BMP2 is an essential target gene.

Forkhead c2 (Foxc2) is a member of the winged spiral transcription factor family. Foxc2 participates in promoting cell proliferation [11, 12], osteogenesis [11, 13–15], and angiogenesis [16]. Moreover, Foxc2 gene-modified mesenchymal stem cells expressed osteo-/odontogenic differentiation-related genes, such as alkaline phosphatase (ALP), osteocalcin (OCN), dentin sialophosphoprotein (DSPP), and dentin matrix protein 1 (DMP1) [11, 13–18]. Latest studies pointed out that Foxc2 was highly expressed in developing dental tissue, including dental papilla [19–21], so it is reasonable to speculate that Foxc2 may participate in the differentiation of SCAP.

In the current research, we constructed Foxc2 and BMP2 gene-modified SCAP by lentivirus transfection technique and then investigated the synergistic effects of Foxc2 and BMP2 in inducing osteogenic and odontogenic differentiation of human SCAP.

## 2. Methods

### 2.1. SCAP Isolation and Identification

A few sound mandibular 3rd molars (*n* = 12) were collected from young patients (18 to 20 years old) in the Oral and Maxillofacial Surgery Department, Sun Yat-sen University. The experiment protocol of the current research was proven by the Ethical Review Committee of Sun Yat-sen University. Freshly extracted third molars were rinsed by PBS, and the apical papillae were isolated by surgical instruments. Sequentially, SCAP were minced and treated with enzymatic digestion according to our previous reports [4, 6]. Osteogenic and adipogenic differentiation of SCAP was analyzed by alizarin red staining and Oil Red staining, respectively. The SCAP phenotypic markers, such as STRO-1 (Santa Cruz, Delaware, CA), CD146 (BD, Pharmingen, USA), CD24 (BD, Pharmingen, USA), and CD45 (BD, Pharmingen, USA), were examined by flow cytometry.

### 2.2. Lentiviral Plasmid Transfection

Human Foxc2 and BMP2 gene primers, as showed in Table 1, were amplified. Then, the amplified oligonucleotides were merged into the blank vector pCDH-CMV-MCS-EF1-copGFP (System Biosciences, USA) to construct lentiviral recombinant plasmids pCDH-Foxc2 and pCDH-BMP2 according to our previous description [4, 6, 22]. Subsequently, the recombinant lentiviral plasmids, envelop plasmid, and packaging plasmid were transduced into 293FT cells. Forty-eight hours after cell culture, the 293FT cell supernatant was collected. The supernatant was used to infect the 3rd passage SCAP to obtain gene-modified cells—that is, SCAP-Foxc2 and SCAP-BMP2. Similarly, SCAP-Foxc2-BMP2 cells were constructed by infecting SCAP with the Foxc2 lentiviral supernatant and BMP2 lentiviral supernatant. SCAP labelled with GFP (SCAP-GFP) was used as the control. Four days after transfection, the expressions of Foxc2 and BMP2 in the four groups, that is, SCAP-GFP, SCAP-Foxc2, SCAP-BMP2, and SCAP-Foxc2-BMP2, were evaluated by RT-qPCR and western blot analysis.

### 2.3. Cell Proliferation

Four group cells were seeded on 96-well plates with initial density of 2 × 10^3^ cells/well and then cultured with *α*-MEM medium supplied with 10% FBS. Cell Counting Kit 8 (CCK8, Dojindo, Tokyo, Japan) was applied to analyze the proliferation of those cells on the 1st, 2nd, 4th, and 8th days after transfection.

### 2.4. RT-qPCR Analysis

Total RNA was extracted by TRIzol (Invitrogen, USA) from the four group cells on the 1st, 4th, 8th, and 16th days after lentiviral transfection. The cDNA synthesis was performed by Revert Aid first strand cDNA synthesis kit (Invitrogen, USA). RT-qPCR reactions were conducted by iQ SYBR Green Supermix (BioRad Laboratories, USA) and regulated by spectrofluorimetric iQ5 thermal iCycler (BioRad Laboratories, USA). The PCR primer sequences were presented in Table 1. The mRNA expressions of Foxc2, BMP2, ALP, OCN, DSPP, and DMP1 in the four group cells were tested by RT-qPCR at different time points. GAPDH was set as the control.

### 2.5. Western Blot

First, proteins were extracted from the four group cells, and then proteins were separated by 12% sodium dodecyl sulfate polyacrylamide gels (Beyotime Institute of Biotechnology, China). The separated proteins were then transferred onto PVDF membranes (Thermo Fisher Scientific, USA) at 200 mA for 2 hours. The 5% nonfat milk was used to block the PVDF membranes for 2 hours. Subsequently, the PVDF membranes were incubated with the primary antibodies overnight at 4°C. The primary antibodies include rabbit polyclonal anti-foxc2 (1 : 500; cat. number ab24340; Abcam, USA), rabbit polyclonal anti-BMP2 (1 : 500; cat. number ab14933; Abcam), and rabbit polyclonal anti-dentin sialoprotein (DSP; 1 : 500; cat. number sc-33586; Santa Cruz Biotechnology, USA). After that, the PVDF membranes were incubated with a horseradish peroxidase-conjugated goat anti-rabbit antibody (1 : 20,000; cat. number ab97051; Abcam) at 37°C for 2 hours. The rabbit polyclonal anti-GAPDH (1 : 2500; cat. number ab9485; Abcam) was chosen as the control. The chemiluminescence western blotting detection system (EMD Millipore) was used to visualize the resultant bands.

### 2.6. ALP and Alizarin Red Staining

The four group cells were seeded on 6-well plates with the density of 5 × 10^4^ cells/well. ALP kit (Jiancheng Biotech, Nanjing, China) and alizarin red kit (Jiancheng Biotech, Nanjing, China) were applied to the cells to visualize ALP expression and mineralized granules on the day 16.

### 2.7. Statistical Analysis

One-way ANOVA test was performed to analyze the differences of the four group cells by SPSS 20.0. Statistical significance was set as *P* value < 0.05.

## 3. Results

### 3.1. Characterization of SCAP

**Figure 1:**
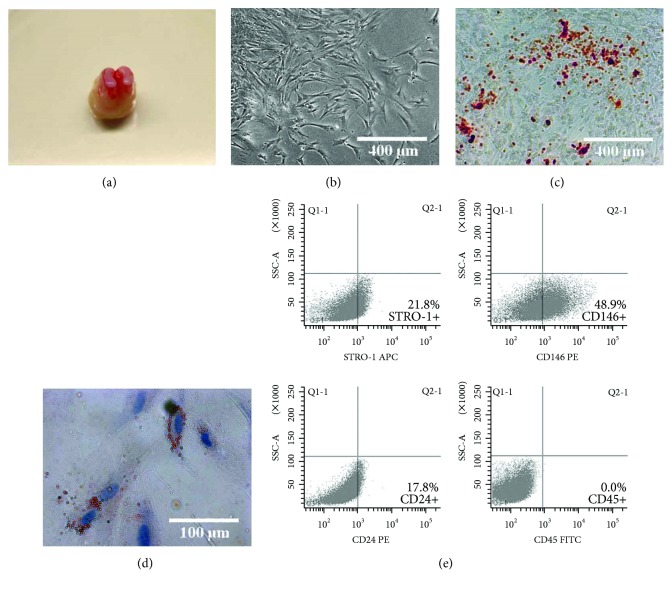
SCAP isolation, culture, and identification. (a) The dental papillae were located at the apical part of the molars and showed pink in color. (b) The primary SCAP were isolated from the dental papilla tissues and displayed spindle-like morphological characters. (c) The 3rd passage SCAP were induced with osteogenic induction medium for 32 days and showed alizarin red-positive mineralized nodules. (d) The 3rd passage SCAP were induced with adipogenic induction medium for 16 days and showed oil red-positive lipid droplets. (e) The 3rd passage SCAP were analyzed by flow cytometry. Those SCAP presented positive expression of STRO-1, CD146, and CD24, but the expression of CD45 was negative.

The papillae, as showed in Figure 1(a), were pink and kidney-shaped. Those primary SCAP isolated from the apical papillae presented classic cell colonies eight days after culture, and most of the SCAP were spindle-like in shape (Figure 1(b)). SCAP formed many alizarin red-positive mineralized nodules after thirty-two days of osteogenic induction (Figure 1(c)) and formed a few oil red O-positive lipid droplets sixteen days after adipogenic induction (Figure 1(d)). The 3rd passage SCAP displayed positive phenotypic markers, such as STRO-1, CD146, and CD24, while CD45 is negative (Figure 1(e)).

### 3.2. Expression of Foxc2 and BMP2 in the Gene-Transfected SCAP

**Figure 2:**
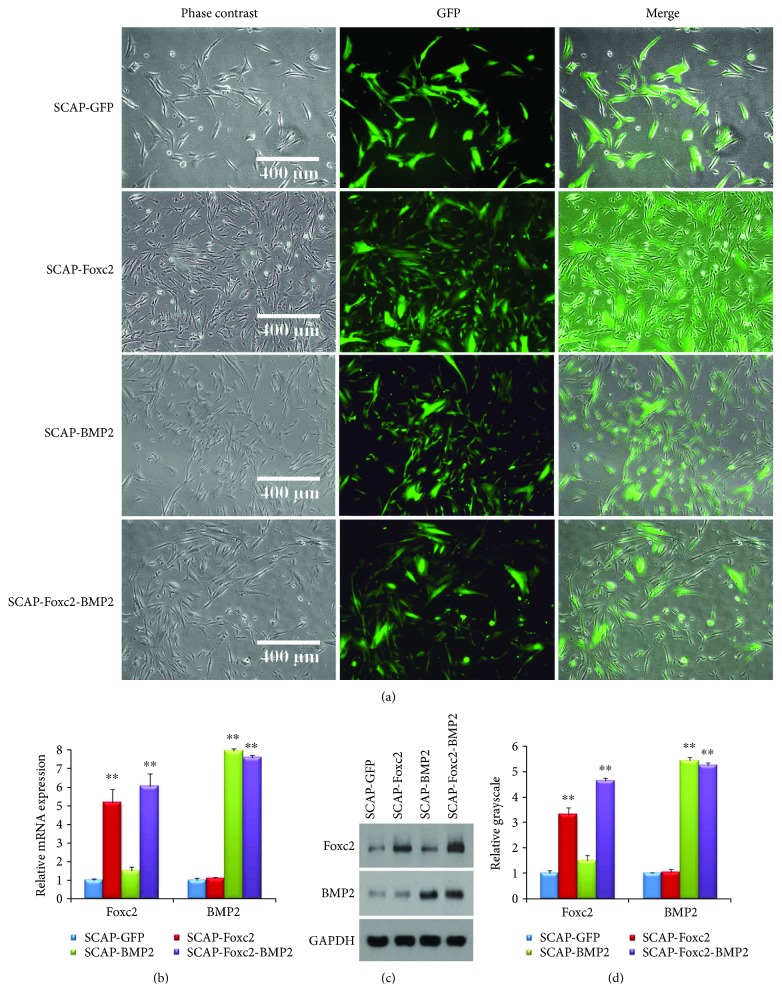
Lentiviral transfection efficiency. (a) Forty-eight hours after lentiviral transfection, all four group cells presented more than 90% GFP expression percentage, which means the lentivirus plasmid-mediated gene transfection efficiencies of the four groups cells are high. (b) Four days after transfection, the Foxc2 expression in the mRNA level was significantly upregulated (*P* < 0.01) in SCAP-Foxc2 and SCAP-Foxc2-BMP2. Similarly, the BMP2 expression in the mRNA level was significantly upregulated (*P* < 0.01) in SCAP-BMP2 and SCAP-Foxc2-BMP2. (c, d) Four days after transfection, western blot results further confirmed that the Foxc2 expression in the peptide level was correspondingly increased (*P* < 0.01) in SCAP-Foxc2 and SCAP-Foxc2-BMP2. The BMP2 expression in the peptide level was also significantly (*P* < 0.01) increased in SCAP-BMP2 and SCAP-Foxc2-BMP2. ^∗∗^*P* < 0.01.

The cells in the four groups showed very high GFP expression percentage four days after transfection (Figure 2(a)). The relative mRNA expression of Foxc2 in SCAP-GFP, SCAP-Foxc2, SCAP-BMP2, and SCAP-Foxc2-BMP2 was 1.00 ± 0.05, 5.14 ± 0.72, 1.52 ± 0.18, and 6.13 ± 0.69, respectively, while the relative mRNA expression of BMP2 in the four group cells was 1.00 ± 0.13, 1.11 ± 0.05, 7.94 ± 0.12, and 7.60 ± 0.10, respectively (Figure 2(b)). The relative Foxc2 peptide expression in the four group cells was 1.00 ± 0.11, 3.29 ± 0.27, 1.52 ± 0.16, and 4.63 ± 0.10, respectively. The relative BMP2 peptide expressions in the four group cells were 1.00 ± 0.05, 1.07 ± 0.11, 5.42 ± 0.15, and 5.23 ± 0.10, respectively (Figures 2(c) and 2(d)). Interestingly, current results presented that BMP2 gene transfection slightly enhanced the mRNA and peptide expression of Foxc2, but the enhancement was not significant (*P* > 0.05).

### 3.3. Foxc2 and BMP2 Regulate the Proliferation of SCAP

**Figure 3:**
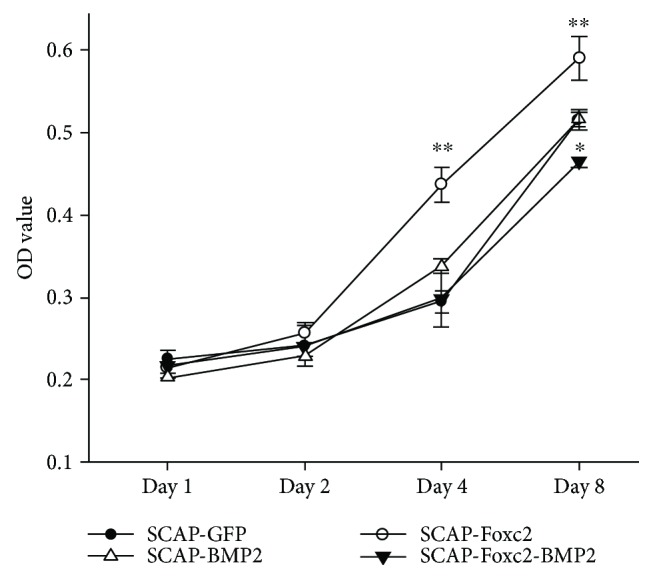
Proliferation status of four group cells. SCAP-Foxc2 demonstrated a significantly higher (*P* < 0.01) proliferation status than the other three group cells on day 4 and day 8, while the proliferation status of SCAP-Foxc2-BMP2 was significantly weaker (*P* < 0.05) than that of the other three group cells on day 8. ^∗^*P* < 0.05, ^∗∗^*P* < 0.01.

As showed in Figure 3, there were no significant differences of the four group cells on the 1st and 2nd days after lentiviral-mediated gene transfection. On the 4th and 8th days, SCAP-Foxc2 showed the highest proliferation level in the four groups (*P* < 0.05). On the 8th day, SCAP-Foxc2-BMP2 displayed the weakest proliferation status (*P* < 0.05).

### 3.4. Foxc2 Potentiated BMP2-Induced Osteo-/Odontogenic Differentiation of SCAP

**Figure 4:**
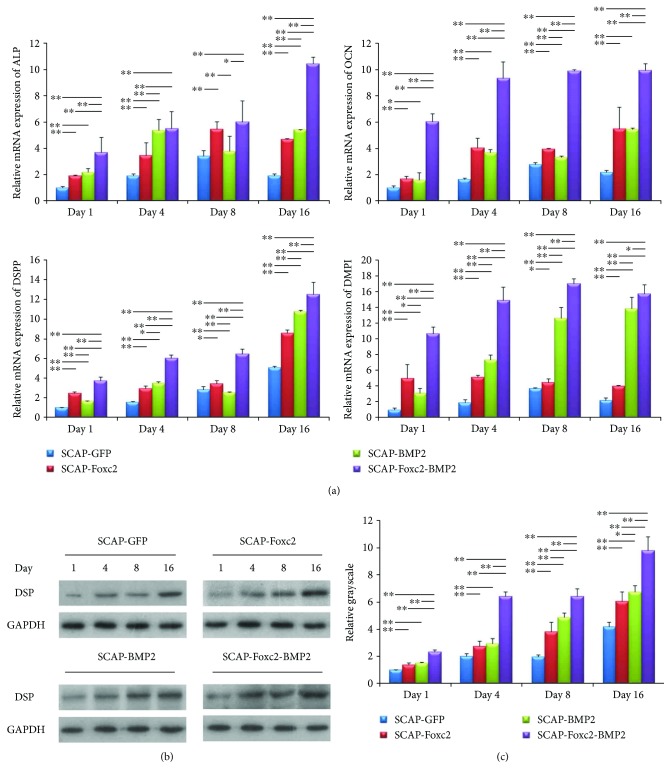
Relative expression of osteo-/odontogenic differentiation of mRNAs and proteins in the four group cells. (a) In SCAP-Foxc2-BMP2, the mRNA expression of ALP, OCN, DSPP, and DMP1 was significantly higher than that of the other groups at majority of the time points (*P* < 0.05). Specifically, the expression exhibited a rising trend over time. (b, c) The DSP expression in SCAP-Foxc2-BMP2, which was detected by relative grayscale of western blot results, was significantly higher than that in the other groups at most of the time points (*P* < 0.05). The expression of DSP in SCAP-Foxc2-BMP2 also displayed a rising trend over time. ^∗^*P* < 0.05, ^∗∗^*P* < 0.01.

The relative mRNA expression of ALP, OCN, and DSPP in SCAP-Foxc2-BMP2 showed a rising trend and achieved the peak expression on day 16 after transfection; however, the expression of DMP1 achieved the peak expression on day 8. Moreover, the mRNA expressions of ALP, OCN, DSPP, and DMP1 in SCAP-Foxc2-BMP2 were significantly higher than those in other groups at most of the time points (*P* < 0.05) (Figure 4(a)). Western blot also showed that the protein expression of DSP in SCAP-Foxc2-BMP2 was significantly higher than that in the other three groups at the four time points (*P* < 0.01) (Figures 4(b) and 4(c)).

### 3.5. ALP Staining of the Gene-Modified SCAP

**Figure 5:**
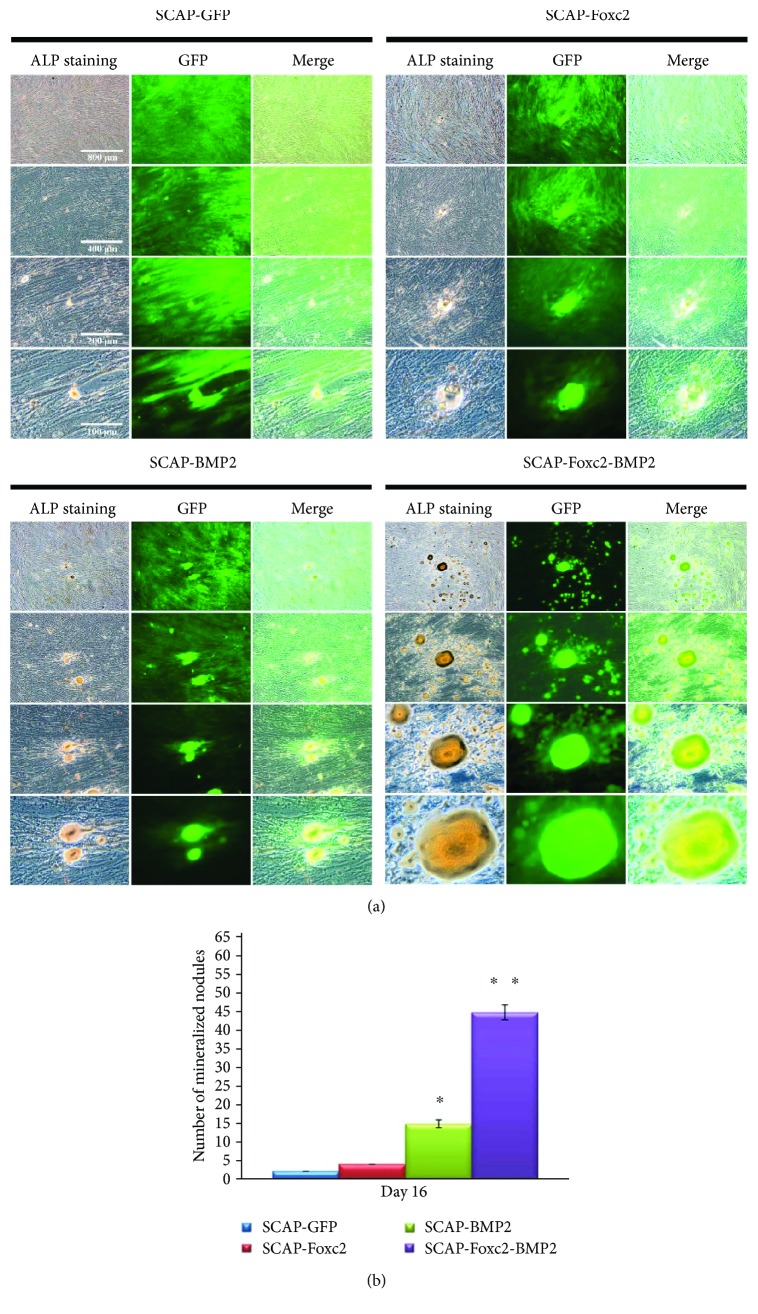
ALP staining status. (a) Those ALP-positive mineralized granules were stained as gold in color. (b) Those mineralized nodules formed by SCAP-Foxc2-BMP2 were significantly more than those formed by the other groups (*P* < 0.01). The mineralized nodules formed by SCAP-BMP2 was significantly more than those formed by SCAP-foxc2 and SCAP-GFP (*P* < 0.05), and mineralized nodules formed by SCAP-Foxc2 was slightly more than those formed by SCAP-GFP. ^∗^*P* < 0.05, ^∗∗^*P* < 0.01.

These mineralized nodules with positive ALP expression were gold in color (Figure 5(a)). The number of mineralized nodules in SCAP-Foxc2-BMP2 was significantly more than that in the other three groups (*P* < 0.01). The number of mineralized nodules formed by SCAP-BMP2 was significantly more than that formed by SCAP-foxc2 and SCAP-GFP (*P* < 0.05) (Figure 5(b)). The number of mineralized nodules of SCAP-Foxc2 was slightly more than that of SCAP-GFP, but there was no statistical significance between the two groups.

### 3.6. Alizarin Red Staining of the Gene-Modified SCAP

**Figure 6:**
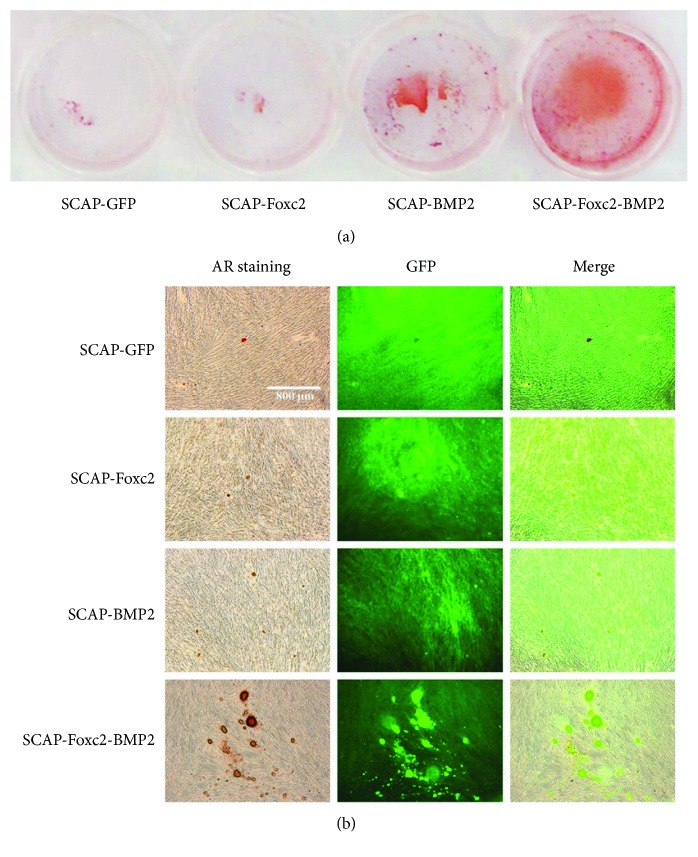
Alizarin red staining status. (a) The mineralized nodules were stained as red. (b) The positive staining area and mineralized nodules' number of SCAP-Foxc2-BMP2 were significantly better than those of the other three group cells.

The mineralized nodules were stained as red (Figure 6(a)). The positive staining area and mineralized nodules' number of SCAP-Foxc2-BMP2 were significantly better than those of the other three group cells (Figure 6(b)). The mineralized nodules' number in SCAP-BMP2 was obviously more than that in SCAP-Foxc2, and the mineralized nodules' number in SCAP-Foxc2 was slightly more than that in SCAP-GFP.

## 4. Discussion

Dental apical papilla directly contributes toward the formation of the tooth roots [23]. As a type of multipotential mesenchymal stem cells derived from the apical papilla, SCAP can differentiate into osteoblasts, odontoblasts, adipocytes, and chondrocytes [24]. In the current research, those mesenchymal cells isolated from the apical papilla formed significant alizarin red-positive mineralized nodes and oil red O-positive lipid droplets after induction *in vitro*. Moreover, those isolated cells presented typical phenotype of SCAP [24], which was verified by STRO-1(+), CD146(+), CD24(+), and CD45(−). Based on the above results, the mesenchymal cells isolated from the apical papilla in the current study were confirmed to be SCAP.

Foxc2 gene, which is necessary for mesenchymal-epithelial interactions during craniofacial development [25–27], has been detected in mesodermal and neural crest derivatives [28]. As a mesenchymal tissue derived from neural crest, the dental apical papilla also expressed Foxc2 [19]. Studies pointed out that BMP proteins and osteogenic induction culture medium could significantly increase the expression of Foxc2 in bone mesenchymal stem cells, dental follicle stem cells, skeletal precursor cells, and amniotic fluid-derived mesenchymal stem cells [15, 20, 29, 30]. The study indicates that Foxc2 is a signal of canonical Wnt pathway in mouse P19 embryonal carcinoma cells [31]. In the current study, Foxc2 gene expression in the BMP2-modified SCAP was slightly increased, but upregulated Foxc2 gene expression did not significantly boost the expression of BMP2 in SCAP. This result suggests that Foxc2 expression is regulated by BMP2 in SCAP, and Foxc2 might be a downstream signal of BMP2. Several scientists have investigated the molecular mechanism of Foxc2 in promoting osteogenic differentiation. Gozo et al. [13] found that the overexpression of Foxc2 can upregulate Wnt4 and BMP4 in osteoblasts. Park et al. [11] suggested that the overexpression of Foxc2 stimulated osteogenic differentiation of osteoblasts by increasing the expression level of integrin *β*1. Kim et al. [18] reported that the overexpression of Foxc2 increased bone formation through *β*-catenin pathway. In the current research, the Foxc2 gene-modified SCAP upregulated the expression of osteo-/odontogenic markers, including ALP, OCN, DSPP, and DMP1. Similar phenomenon was also observed in other Foxc2-modified cells, such as bone marrow mesenchymal stem cells [14, 30], myoblasts [13], and osteoblasts [18].

Furthermore, our research presented that lentivirus-mediated Foxc2 gene transfection improved the ability of SCAP to form mineralized nodules *in vitro*, indicating that Foxc2 gene participated in regulating of mineralization process. Additionally, the mineralization capability of SCAP-Foxc2-BMP2 was significantly stronger than that of the other three groups. Mineralized nodules formed by SCAP-Foxc2-BMP2 cells were about 3 times more than those of SCAP-BMP2, about eleven times more than those of SCAP-Foxc2, and about twenty-two times more than those of SCAP-GFP. These results suggest that Foxc2 and BMP2 synergistically promote osteo-/odontogenic differentiation and mineralization of SCAP.

A few studies discovered that Foxc2 could stimulate proliferation of some cells. In myoblasts, decreased endogenous Foxc2 reduced cell proliferation activity, while increased expression of Foxc2 promoted cell proliferation [13]. In osteoblasts, upregulated Foxc2 facilitates cell proliferation *in vitro* [32], and Foxc2 might encourage the survival and proliferation of osteoblasts by upregulating integrin *β*1 [11]. Consistent with the previous reports [13, 32], our data showed that the proliferation of Foxc2 gene-transfected SCAP was slightly stronger than that of the other three groups. However, the proliferative activity of Foxc2 and BMP2 double gene-transfected SCAP was reduced on day 8, which implies that Foxc2 and BMP2 synergistically control the proliferation of SCAP. Some studies pointed out that Foxc2 promoted proliferation by activating mTORC1 in preadipocytes [12] and mesenchymal stem cells [33]. However, in osteoblasts, mTORC1 was activated by BMP2 to promote osteogenic differentiation [34]. So, it is speculated that the function of mTORC1 may be changed from promoting cell proliferation to enhancing cell osteo-/odontogenic differentiation when Foxc2 and BMP2 were both activated in SCAP.

In summary, our research indicated that Foxc2 and BMP2 genes could act synergistically to improve osteo-/odontogenic differentiation and mineralization of SCAP *in vitro*. These results have a positive impact on the clinical practice of tooth regeneration. The future study could focus on the interaction between these two genes and the mechanism of their cooperative function on SCAP. Moreover, *in vivo* studies could further support this discovery.

## Figures and Tables

**Table 1:** Real-time PCR primers.

Genes	Sequence	Size (bp)
BMP2	Forward: 5′-ACCCGCTGTCTTCTAGCGT-3′	180
	Reverse: 5′-TTTCAGGCCGAACATGCTGAG-3′	
Foxc2	Forward: 5′-CCTCCTGGTATCTCAACCACA-3′	134
	Reverse: 5′-GAGGGTCGAGTTCTCAATCCC-3′	
ALP	Forward: 5′-CTATCCTGGCTCCGTGCTC-3′	100
	Reverse: 5′-GCTGGCAGTGGTCAGATGTT-3′	
OCN	Forward: 5′-CTCACACTCCTCGCCCTATT-3′	107
	Reverse: 5′-TTGGACACAAAGGCTGCAC-3′	
DSPP	Forward: 5′-GCCACTTTCAGTCTTCAAAGAGA-3′	130
	Reverse: 5′-GCCCAAATGCAAAAATATGTAA-3′	
DMP1	Forward: 5′-AAAATTCTTTGTGAACTACGGAGG-3′	94
	Reverse: 5′-GAGCACAGGATAATCCCCAA-3′	
GAPDH	Forward: 5′-AAGGTGAAGGTCGGAGTCAA-3′	108
	Reverse: 5′-AATGAAGGGGTCATTGATGG-3′	

## Data Availability

The data used to support the findings of this study are available from the corresponding author upon request.
